# Comparison of Six Data Cleaning Methods for Determining Repetitive Head Impact Exposure in Youth Tackle Football

**DOI:** 10.1007/s10439-026-03991-4

**Published:** 2026-01-23

**Authors:** Samantha DeAngelo, Adam Culiver, Enora Le Flao, Nick Shoaf, Durshil Doshi, Ryan Tracy, Nii-Ayi Aryeetey, Anna Quatrale, Carly Smith, Jianing Ma, Jeff Pan, Jingzhen Yang, Sean C Rose, James Onate, Nathan Edwards, Zeynep Saygin, Jaclyn B. Caccese

**Affiliations:** 1 School of Health and Rehabilitation Sciences, College of Medicine, The Ohio State University Wexner Medical Center, Columbus, OH, USA; 2 Chronic Brain Injury Program, The Ohio State University, Columbus, OH, USA; 3 Department of Physical Therapy, School of Health and Human Sciences, Indiana University Indianapolis, Indianapolis, IN, USA; 4 Academic Health Center, Indiana University Health, Indianapolis, IN, USA; 5 Human Performance Collaborative, The Ohio State University, Columbus, OH, USA; 6 Department of Psychology, College of Arts and Sciences, The Ohio State University, Columbus, OH, USA; 7 Center for Biostatistics, The Ohio State University, Columbus, OH, USA; 8 Center for Injury Research and Policy, Abigail Wexner Research Institute at Nationwide Children’s Hospital, Columbus, OH, USA; 9 Neurology, Nationwide Children’s Hospital, Columbus, OH, USA; 10 246C Atwell Hall, 453 W 10th Ave, Columbus, OH 43210, USA

**Keywords:** Head acceleration events, Sensor validation, Concussion, Video verification

## Abstract

**Purpose:**

Instrumented mouthguards (iMGs) are commonly used to quantify head acceleration event (HAE) exposure, but accurate interpretation requires rigorous data cleaning methods. This study compared six data cleaning methods for determining HAE rates and magnitudes, as well as cleaning method validity compared to the 5^th^ method video verification in youth tackle football.

**Methods:**

Fifty athletes (ages 8–12) wore Impact Monitoring Mouthguards during games across one season. Six data cleaning methods were applied to HAEs, including uncleaned data, time-windowing, proprietary classification algorithms, video verification, and combinations thereof. Impact rate, peak linear acceleration (PLA), and peak rotational velocity (PRV) were compared across methods using rate ratios, and intra-class correlation coefficients (ICCs), and non-parametric analyses.

**Results:**

Data cleaning methods significantly influenced HAE rate but had minimal effect on magnitude. The uncleaned dataset produced the highest HAE rate (67.75 per athlete exposure), while the most stringent method (i.e., time-windowed, proprietary algorithm-classified, video-verified data) yielded the lowest (0.70 per athlete exposure). Although the time-windowed, proprietary algorithm-classified data demonstrated high specificity (0.96), it demonstrated low sensitivity (0.37) and positive predictive value (0.39) when compared to video-verified data. Differences in PLA across methods were not significant; only one significant difference in PRV was observed.

**Conclusions:**

These findings highlight the impact of data cleaning on HAE quantification in youth tackle football. Although video verification remains best practice, it is resource intensive. Time-windowed, algorithm-classified data may serve as an efficient proxy in similar cohorts, though researchers should recognize its limitations. Findings support the need for standardized data cleaning methods and transparent reporting to ensure accurate and comparable HAE exposure estimates.

## Introduction

In the United States, over two million youth athletes participate in tackle football annually [1, 2]. However, overall youth sport participation is declining, with football experiencing the steepest drop [3, 4]. Recently, concerns have grown regarding the potential neurodevelopmental effects of sustaining repetitive head impacts (RHIs) during critical periods of neurodevelopment [5, 6]. While football safety discussions have historically focused on concussions, increasing attention is now being directed toward RHIs, which occur without immediate symptoms or clinical diagnosis. RHIs are a routine part of normal play, raising significant concern about their cumulative effects over time [6].

Studies investigating the effects of RHIs in humans have produced conflicting findings. Some report microstructural brain changes and short-term functional impairments, [7–17] while others find minimal link between RHI exposure and short-term outcomes [18–24]. Similarly, findings on the long-term effects are heterogeneous, and few studies have specifically addressed long-term outcomes in tackle football players who only played at the youth level [25–29]. These discrepancies suggest that factors such as the timing, frequency, and magnitude of RHIs may influence the extent of impairment, [30–32] highlighting the importance of accurately quantifying RHI exposure to better understand its potential impact.

Concerns regarding RHI exposure have led to the adoption of monitoring strategies using wearable sensors. These devices, typically containing accelerometers and gyroscopes, record head acceleration events (HAEs) to estimate the skull–and by extension, brain–movement during RHIs in real time. HAE typically refers to sensor-recorded data and will be used throughout this paper to denote RHIs. Several types of wearable devices have been used in studies, including skin-based sensors, headband-affixed sensors, helmet-affixed sensors, and instrumented mouthguards (iMGs) [33–38]. These wearable devices have undergone both laboratory validation, using controlled impacts on instrumented head forms, and on-field validation, where HAEs are matched with observed impacts on video [35, 37]. In tackle football, the Head Impact Telemetry System (HIT) has been the most widely used system, embedding accelerometers inside helmets [38]. However, proper helmet fit is critical to avoid measuring helmet movement rather than true skull motion—a concern that similarly affects headband- and skin-based sensors [39, 40]. iMGs are worn on the teeth, providing a more direct coupling to the skull [35, 41]. Among available iMGs, Prevent Biometrics’ Impact Monitor Mouthguard has demonstrated similar or better kinematic accuracy and on-field performance compared to other commercially available options [35, 42, 43].

Despite advancements in sensor technology, inaccuracies and inconsistencies in HAE data collection remain a significant challenge. A primary concern is the inclusion of false-positive HAE recordings, where the iMG recordings do not represent true head movement, as this has been shown to over-estimate the count and magnitude of HAEs [40, 44]. To address this, researchers use data cleaning techniques to improve the validity of reported exposures [33, 36, 45, 46]. The Consensus head acceleration measurement practices (CHAMP) guidelines recommend two primary methods: time-windowing and video verification [46]. Time-windowing involves removing HAEs recorded outside of active play periods (e.g., before and after the game, halftime, and timeouts), while video verification involves matching each sensor-recorded HAE to synchronized video footage to confirm its occurrence [46]. Although video verification methods improve data quality, there are inherent limitations in video verification methods and as such, video verification is considered a best practice rather than a gold standard. Additionally, video verification methods are labor-intensive, requiring significant personnel training, time, and effort.

Many commercially available iMGs, use proprietary “black-box” classification algorithms designed to automatically filter out false positive HAEs.[45–48] These algorithms aim to reduce the burden of manual data cleaning by identifying true HAEs without requiring extensive post-processing. However, few studies have rigorously evaluated the accuracy of these algorithms compared with manual methods.[32, 49] As the use of wearable devices becomes increasingly common, establishing standardized, efficient, and valid data cleaning procedures is critical for enabling consistent comparisons of HAE exposure across studies.

The primary purpose of this study was to evaluate the validity of six common data cleaning methods for iMG-measured HAEs in youth tackle football, using video verification as the criterion measure. A secondary objective was to investigate the downstream effects of each method on HAE rate and magnitude to understand how data validity influences these common outcome measures of HAE exposure. We hypothesized that data cleaning method would affect validity, rate, and magnitude.

## Materials and Methods

### Study Design

This was a prospective observational study.

### Participants

Athletes from a single American youth tackle football program (3rd-6th grade) were invited to participate in the study. A total of 67 athletes aged 8 to 12 years enrolled in the study, but 8 dropped out due to iMG discomfort, our primary analysis included 50 total athletes. All participants provided written assent and parents provided written parental informed consent. This study was approved by The Ohio State University Institutional Review Board.

### Instrumentation

Participants wore either custom-fit (*n* = 50) or boil-andbite (*n* = 9) Impact Monitoring Mouthguard (V2; Prevent Biometrics, Edina, MN). Custom-fit iMGs were made from 3D scans obtained using an intraoral scanner (i-Tero, AZ, USA), while boil-and-bite iMGs were fit by placing the iMG in boiling water for 20 seconds, aligning the iMG to the teeth and allowing it to set in the mouth for 30 seconds, followed by a cold-water rinse.[50] Athletes who had braces (*n* = 7) or were not present when 3D scans were obtained (*n* = 2) wore boil-and-bite iMGs, all other athletes used custom-fit iMGs. Due to resulting data discrepancies observed in the boil-and-bite group, particularly among athletes with braces, likely due to poor fit, only data from the custom-fit iMGs are included in the primary analysis (*n* = 50). The data from the total sample (*n* = 59) and from only boil-and-bite iMGs (*n* = 9) are provided for comparison in the Supplementary Material.

Each iMG was equipped with a tri-axial accelerometer and gyroscope, both sampling at 3200 Hz, capable of measuring linear acceleration up to 200 *g* per axis and rotational velocity up to 35 rad/s per axis. The iMGs triggered recording when linear acceleration exceeded 8 *g* on any single axis and recorded 10 ms before and 40 ms after the trigger event. All impacts were processed through Prevent Biometrics’ proprietary algorithm (Software Version 2.1.24–38), which includes filtering, transformation to the head center of gravity, and correction factor application based on the algorithm-determine quality class. An inclusion threshold on the processed peak linear acceleration (PLA) of 10 *g* was utilized in this study, consistent with common practice in sports research to distinguish impact-HAEs from non-impact events.[51, 52] The PLA and peak rotational velocity (PRV) provided by the manufacturer were used for all analyses.

### Data Collection Sessions

Participants wore iMGs during the 2023 season for one to eight games. Although athletes practiced together, games were played separately by three grade-based teams (i.e., 3^rd^/4^th^ grade, 5^th^ grade, and 6^th^ grade), totaling 20 games. A research team member attended each game to distribute and collect iMGs, video record the game, monitor iMG compliance, and maintain device functionality, including charging, sanitization, and synchronization with video. Athlete attendance was documented to calculate athlete exposures, defined as one athlete participating in one game while wearing a functioning iMG. Proper iMG wear during games was monitored by the on-field referees as part of league standards.

### Video Recording

All games were video recorded with a Sony 4k Cyber-Shot RX100 camera (Sony, Tokyo, Japan) 50 frames/s and a resolution of 1080 p from the best vantage point at each field. At the start of each recording, an iPhone clock (Apple, Cupertino, CA) was displayed to time-synchronize the video and iMG data collected through the Prevent Biometrics app. Video recordings were used for both time-windowing (i.e., recording active play periods and removing breaks such as halftime and timeouts) and for guided video verification of HAEs.

### RHI Data Synchronization

We synchronized video recordings with IMG data using a two-step process. During data collection, a phone displaying the world clock, with a 1 minute resolution, was displayed on video. In the first step, this was used to coarsely align the HAE timestamps on the video timeline. In the second step, we identified a specific head impact that was both recorded by the IMG and clearly visible on the video footage. This impact served as a “ground truth” event, and was used to recalculate the precise alignment between the HAE timestamps and the video timeline.

### Data Cleaning & Analysis

Six data cleaning methods were applied to iMG data (Table 1). For all Methods only iMG recorded HAEs over the 10 *g* inclusion threshold were included (visually identifiable HAEs that did not trigger iMG recordings were not included). For each data cleaning method, impact count and impact rate (impact count/athlete exposure) were calculated. To compare rates, methods were compared using impact rate ratios (Method A Impact Rate/Method B Impact Rate), with corresponding 95% confidence intervals (CIs), and two-way mixed-effects intraclass correlation coefficients (ICCs) for absolute agreement [53, 54]. Impact rate ratios with 95% CIs not containing 1.0 were considered statistically significant. ICCs were interpreted as follows: < 0.5 indicated poor agreement, between 0.5 and 0.75 indicated moderate agreement, between 0.75 and 0.9 indicated good agreement, and > 0.9 indicated excellent agreement. To establish validity, sensitivity, specificity, and positive predictive value (PPV) were calculated for all methods with Method 5 (video verification) as the reference. This was done to compare the current best practice of video verification to the other presented cleaning methods.[55] True positives were defined as HAEs classified as true by both Method 5 (video verification) and the compared method. False positives were defined as HAEs classified as false by Method 5 but true by the compared method. False negatives were defined as HAEs classified as true by Method 5 but false by the compared method. True negatives were defined as HAEs classified as false by both Method 5 and the compared method. Differences in impact magnitudes (i.e., player median PLA and median PRV) across methods were analyzed using a Related-Samples Friedman’s Two-Way ANOVA with significance adjusted using a Bonferroni correction.

Inter-rater reliability for video verification was determined from six games independently reviewed by two raters. Across the six games, Rater 1 classified 124 HAEs and Rater 2 classified 167 HAEs as true positive impacts, resulting in excellent agreement, with an ICC of 0.950 (95% CI: 0.902–0.975; *p* < 0.001). Out of 4921 recorded HAEs, 100 were identified as true HAEs by both raters, while 91 resulted in discrepancy between raters.

## Results

Data from 50 athletes whose custom-fit iMGs successfully recorded HAEs during the season were included in the analysis (9 athletes who had boil-and-bite iMGs are included in the Supplementary Material; total *n* = 59, mean age = 10.2 ± 1.0 years, height = 145.7 ± 10.7 cm, mass = 40.8 ± 9.1 kg). Across the 50 athletes, a total of 232 athlete-exposures were recorded across 20 games, with athletes participating in one to eight games each. Among the six data cleaning methods, Method 6 (time-windowed, HAEs confirmed by both proprietary algorithm and video verification) resulted in the lowest HAE count and rate per athlete exposure (Table 2). Impact rate ratios differed significantly across all data cleaning methods except between Methods 4 (time-windowed, algorithm-classified HAEs) and 5 (video verification) (Table 2). The smallest difference was observed between Methods 4 and 5, while the largest difference was observed between Methods 1 (uncleaned) and 6. ICCs indicated good agreement (0.836) between Method 3 (algorithm-classified HAEs without time windowing) and 4. All other ICCs indicated less than or equal to moderate agreement (Table 2).

Sensitivity, specificity and PPV results are presented in Table 3. Sensitivity, specificity and PPV were calculated with Method 5 as the reference criteria. Methods 1, 2 and 6 resulted in the highest sensitivity value of 1, while methods 3 and 4 resulted in lower sensitivity (0.37). Method 3 resulted in the highest specificity value (0.97), followed by method 4 (0.96). For PPV, method 4 resulted in the highest value (0.39), followed by method 3 (0.26).

No significant differences in PLA were observed across data cleaning methods (Table 4). For PRV, only one significant difference was observed, between Method 1 and Method 3 (Table 5); all other pairwise comparisons were not statistically significant.

## Discussion

The primary purpose of this study was to evaluate the validity of several common data cleaning methods for iMG-measured HAEs in youth tackle football, using video verification as the criterion measure. A secondary objective was to investigate the downstream effects of each method on HAE rate and magnitude to understand how data validity influences these common outcome measures of HAE exposure. Partially consistent with our hypothesis, the data cleaning method significantly influenced HAE validity and rate but had minimal effects on HAE magnitudes.

Overall, results for the measures of validity (sensitivity, specificity and PPV) varied based on data cleaning method. Method 1 (uncleaned) represented the full dataset and all subsequent Methods contained HAEs included in the Method 1 dataset. Not surprisingly, Methods 1 and 2 resulted in very high sensitivity but very low specificity and PPV, recording all HAEs and not filtering out false positives. In contrast, Methods 3 and 4 resulted in high specificity but had relatively lower sensitivity and PPV. These findings suggest that although the proprietary algorithm effectively filters out false positives, it also misclassifies a substantial proportion of true HAEs in our cohort of athletes. Prior work has shown that between 14 and 40% of true HAEs may be incorrectly discarded by iMG algorithms, [35, 47, 52–54] particularly when iMGs are poorly coupled to the teeth. Inadequate coupling reduces the quality of the biomechanical recordings, increasing the likelihood that true HAEs are filtered out by the proprietary algorithm [37]. Specifically, Method 4 was identified by our research team as a time-efficient method compared to video verification however, our results suggest relying solely on algorithm-classified data risks underestimating exposure. When feasible, video verification remains essential for ensuring accurate HAE counts which is currently in accordance with the CHAMP guidelines [46].

A secondary aim was to quantify the extent to which HAE rates vary across methods. We used rate ratios to examine relative differences in HAE rates (i.e., group-level proportional differences), while ICCs assessed the consistency of exposure rankings across methods (i.e., individual-level consistency). Because these metrics capture different aspects of agreement, discrepancies between them were expected. We observed significant differences in both impact rate and impact rate ratio across all data cleaning methods, except for Methods 4 (algorithm-classified HAEs with time windowing) and 5 (video verification), suggesting that time-windowed, algorithm-classified methods may serve as a practical proxy when resources are limited. However, researchers should interpret findings with caution and acknowledge potential limitations, as noted above. As expected, Method 1 (uncleaned) resulted in the highest HAE count and impact rate, whereas Method 6 (HAEs confirmed by both proprietary classification algorithm and video verification) yielded the lowest. Notably, Method 2 (time-windowed HAEs only) resulted in approximately 15 times more HAEs than Method 5, reinforcing prior findings that time-windowing alone substantially overestimates true exposure compared to video verification, likely due to false positive HAEs occurring during active game play [53, 56–59]. ICCs results represent good agreement between Method 3 (algorithm-classified HAEs without time windowing) and Methods 4. These results suggest that while these methods may not agree on exact HAE counts, they are consistent in identifying which athletes experienced the most exposure.

In contrast to HAE rate, HAE magnitude (i.e., PLA and PRV) was surprisingly consistent across the data cleaning methods, a finding that contradicted our hypothesis that methods retaining more false positives would yield significantly inflated values [40, 44]. While a slight trend of higher magnitudes in the least restrictive methods (1 and 2) was observed, the general lack of statistical significance carries a critical implication that magnitude distributions are not reliable indicators of overall dataset validity. Although a combined approach like Method 6, which uses both video and biomechanical criteria, likely provides the most accurate PLA and PRV values for confirmed true positive events, a dataset contaminated with false positives can still present a statistically similar magnitude profile to a clean one. This underscores the danger of relying on outcome measures to assess data quality, especially if HAE magnitude is being considered separately from HAE rate.

This study has several limitations. First, our sample included only youth tackle football players aged 8–12 years, limiting generalizability to other age groups or sports. Collegiate tackle football athletes typically sustain more frequent and higher-magnitude HAEs, [60] so data cleaning method effects may differ. Second, only one HAE monitoring system (i.e., Prevent Biometrics) and one version of the device and procession algorithm were used; results may not apply to other devices or past and future versions of the algorithms. For our primary analysis, we excluded athletes who wore boil-and-bite iMGs due to data discrepancies for athletes with braces. We have included both the full (*n* = 59) and the boil-and-bite only (*n* = 9) results in the Supplementary Material. Of all 2398 boil-and-bite iMG-recorded HAEs, only 2 HAEs were deemed true positives by the proprietary-algorithm. This suggests that braces highly impact the outcomes of the proprietary algorithm classification, therefore the validity of the data from athletes with braces may be compromised. Additionally, we recognize the potential limitations of choosing a 10g inclusion threshold which may mis-represent some contact versus non-contact events. We chose this cutoff due to its common use as an impact threshold in the literature [52]. Device malfunction and athlete attrition contributed to data loss, which limited the athlete exposures available for analysis. Furthermore, we acknowledge that iMGs stand the potential to underestimate HAE counts if HAEs fail to meet PLA thresholds or due to sensor error [61–63]. Because we did a guided video verification process, our data set does not include false negative HAEs that were not recorded by the iMGs. Additionally, although video verification is considered the current best practice, there are inherent limitations of this method, such as issues with camera placement, field of view, and rater variability. Our single-camera setup contrasts with CHAMP recommendations for multi-angle views.[46] Distinguishing true from false positive impacts based solely on video footage can introduce uncertainty. Despite excellent inter-rater reliability, we observed a 91-HAE difference between raters over 6 games, highlighting challenges with video review [46]. We have attempted to mitigate discrepancies moving forward through improvement in training techniques and increased practice before starting the video verification process.

In conclusion, data cleaning methods have a substantial impact on HAEs rate but a limited effect on median HAE magnitude in youth tackle football games. When comparing to the current best practice of video verification, the Prevent Biometrics’ proprietary algorithm combined with time windowing demonstrated high specificity but low sensitivity and PPV. Although this method represents an efficient approximation, video verification likely remains the most accurate method for validating exposure. These findings highlight the importance of transparent reporting and standardization of HAE data cleaning methods to ensure comparability across studies utilizing iMGs.

## Supplementary Material

Supplementary material

## Figures and Tables

**Table 1:** Description of six data cleaning methods analyzed

Method Number	Description
Method 1	All HAEs without cleaning, regardless of classification or timing.
Method 2	Time-windowed HAEs limited to active game play, excluding periods before the start of the game, after the end of the game, and during breaks (e.g., halftime, time outs).
Method 3	HAEs classified as true positives by prevent biometrics’ proprietary classification algorithm without time-windowing.
Method 4	Time-windowed HAEs classified as true positives by Prevent Biometrics’ proprietary classification algorithm.
Method 5	Video verification, where a trained research team member performed a guided video review of all time-windowed HAEs recorded by the IMGs in Sportscode (Hudl, Lincoln, NE) and coded HAEs as true or false positive based on observable contact synchronized to the recorded game footage. Only iMG recorded HAEs were included (visually identifiable HAEs that did not trigger iMG recordings were not included). HAEs recorded by iMGs that occurred outside of the camera view (*n* = 90) and could not be verified were excluded from analyses.
Method 6	Time-windowed, HAEs classified as true positives by both the Prevent Biometrics’ proprietary classification algorithm and confirmed through video verification.

**Table 2:** Comparison of head acceleration event (HAE) exposure across six data cleaning methods

Method	Impact number	Impact rate (95% CI)	Compared method	Impact rate ratio (95% CI)	ICC (95% CI)
1 (Uncleaned)	15719	67.75 (59.52–77.13)	2	2.36 (2.29–2.43)*	0.632 (0.381–0.786)*
			3	25.23 (23.29–27.33)*	0.008 (− 0.205–0.242)
			4	37.43 (33.97–41.23)*	0.004 (− 0.207–0.238)
			5	35.40 (32.22–38.91)*	0.010 (− 0.202–0.244)
			6	96.44 (82.65–112.53)*	0.001 (− 0.208–0.234)
2 (time-windowed HAEs only)	6666	28.73 (25.21–32.75)	3	10.70 (9.86–11.62)*	0.012 (− 0.205–0.249)
			4	15.87 (14.38–17.52)*	0.009 (− 0.205–0.243)
			5	15.01 (13.64–16.53)*	0.024 (− 0.191–0.259)
			6	40.90 (35.01–47.77*	0.002 (− 0.208–0.234)
3 (algorithm-classified HAEs without time windowing)	623	2.69 (2.31–3.12)	4	1.48 (1.31–1.68)*	0.836 (0.612–0.921)*
			5	1.40 (1.24–1.58)*	0.424 (0.175–0.623)*
			6	3.82 (3.22–4.54)*	0.284 (− 0.002–0.525)*
4 (algorithm-classified HAEs with time windowing)	420	1.81 (1.54–2.13)	5	0.95 (0.83–1.08)	0.505 (0.271–0.684)*
			6	2.58 (2.15–3.09)*	0.476 (0.142–0.693)*
5 (video verification)	444	1.91 (1.63–2.24)	6	2.72 (2.28–3.26)*	0.386 (0.003–0.645*
6 (HAEs confirmed by both proprietary classification algorithm and video verification)	163	0.70 (0.58–0.86)			

Impact count and impact rate per athlete exposure are presented for each data cleaning method. Impact rate ratios (Impact rate for method A divided by impact rate for method B and intraclass correlation coefficients (ICCs) with 95% confidence intervals *CI* were calculated to assess agreement between methods. ICCs were interpreted as follows: < 0.5 = poor agreement, 0.5–0.75 = moderate agreement, 0.75–0.90 = good agreement, > 0.90 = excellent agreement.

* indicates a significant difference was observed. *HAE* head acceleration event

**Table 3:** Measures of validity across four data cleaning methods with Method 5 (video verification) as the criterion

Method compared to method 5 (Video-verification)	Sensitivity	Specificity	Positive predictive value (PPV)
1 (Uncleaned)	1.00	0.00	0.03
2 (time-windowed HAEs only)	1.00	0.60	0.07
3 (algorithm-classified HAEs without time windowing)	0.37	0.97	0.26
4 (algorithm-classified HAEs with time windowing)	0.37	0.96	0.39
6 (HAEs confirmed by both proprietary classification algorithm and video verification)	1.00	N/A	1.00

Sensitivity, specificity and positive predictive value (PPV) are presented for methods one through six. Method 5 (video-verification) is used as the reference criteria

**Table 4:** Comparison of peak linear acceleration (PLA) across six data cleaning methods

Peak linear acceleration (PLA, g)					
Method	Median	25^th^ percentile	75^th^ percentile	Compared method	Corrected *p*-value
1 (Uncleaned)	14.6	13.6	16.7	2	1.000
				3	1.000
				4	1.000
				5	1.000
				6	1.000
2 (time-windowed HAEs only)	15.0	13.5	16.0	3	1.000
				4	1.000
				5	1.000
				6	1.000
3 (algorithm-classified HAEs without time windowing)	13.9	12.7	16.7	4	1.000
				5	1.000
				6	1.000
4 (algorithm-classified HAEs with time windowing)	13.9	12.6	15.9	5	1.000
				6	1.000
5 (video verification)	14.3	12.8	17.1	6	1.000
6 (HAEs confirmed by both proprietary classification algorithm and video verification)	14.4	11.9	15.9		

Median peak linear acceleration (PLA) values are presented for each data cleaning method. Differences in PLA across methods were assessed using a related-samples Friedman’s Two-Way ANOVA with significance values adjusted by a Bonferroni correction

**Table 5:** Comparison of peak rotational velocity (PRV) across six data cleaning methods

Peak rotational velocity (PRV, rad/s)					
Method	Median	25th percentile	25th percentile	Compared method	Corrected *p*-value
1 (uncleaned)	8.5	6.5	10.1	2	1.000
				3	0.003 *
				4	0.268
				5	1.000
				6	1.000
2 (time-windowed HAEs only)	8.5	6.2	9.8	3	0.063
				4	1.000
				5	1.000
				6	1.000
3 (algorithm-classified HAEs without time windowing)	6.5	4.8	9.4	4	1.000
				5	0.449
				6	0.672
4 (algorithm-classified HAEs with time windowing)	7.2	5.3	10.0	5	1.000
				6	1.000
5 (video verification)	7.2	6.0	10.4	6	1.000
6 (HAEs confirmed by both proprietary classification algorithm and video verification)	7.4	5.6	8.8		

Median peak rotational velocity (PRV) values are presented for each data cleaning method. Differences in PRV across methods were assessed using a related-samples Friedman’s Two-Way ANOVA with significance values adjusted by a Bonferroni correction.

* indicates a significant difference was observed

## Data Availability

Datasets generated and analyzed during the current study will be available in the DASH repository.
