# Supplementary material for: Comparison of Six Data Cleaning Methods for Determining Repetitive Head Impact Exposure in Youth Tackle Football
Source: Ann Biomed Eng. Author manuscript; Available in PMC 2026 Mar 21. (PMC13004607; doi:10.1007/s10439-026-03991-4)
Supplement: Supplementary file 1 [file NIHMS2154539-supplement-Supplementary_material.pdf]

Supplementary Material

The following tables (S1-S4) include all 59 athletes (boil-and-bite n=9, custom-fit n=50).

Table S1. Comparison of head acceleration event (HAE) exposure across six data cleaning methods.

Method	Impact Number	Impact Rate (95% CI)	Compared Method	Impact Rate Ratio (95% CI)	ICC (95% CI)
1 (Uncleaned)	18117	65.17 (57.89-73.36)	2	2.41 (2.35-2.47) *	.678 (0.512-0.794) *
			3	28.99 (26.77-31.39) *	.053 (-0.188-0.293)
			4	42.93 (38.98-47.28) *	.035 (-0.205-0.276)
			5	36.16 (33.09-39.52) *	.046 (-0.195-0.286)
			6	110.47 (94.73-128.83) *	.014 (-0.225-0.256)
2 (time-windowed HAEs only)	7520	27.05 (24.00-30.49)	3	12.03 (11.09-13.05) *	.132 (-0.112-0.365)
			4	17.82 (16.16-19.66) *	.088 (-0.155-0.325)
			5	15.01 (13.71-16.43) *	.115 (-0.128-0.350)
			6	45.85 (39.28-53.53) *	.034 (-0.206-0.275)
3 (algorithm-classified HAEs without time windowing)	625	2.25 (1.95-2.59)	4	1.48 (1.31-1.68) *	.917 (0.862-0.950) *
			5	1.25 (1.11-1.40) *	.973 (0.955-0.984) *
			6	3.81 (3.21-4.53) *	.468 (0.249-0.643) *
4 (algorithm-classified HAEs with time windowing)	422	1.52 (1.30-1.77)	5	0.84 (0.74-0.96) *	.952 (0.920-0.971) *
			6	2.57 (2.15-3.08) *	.660 (0.489-0.783) *
5 (video verification)	501	1.80 (1.56-2.09)	6	3.05 (2.56-3.64) *	.533 (0.327-0.691) *
6 (HAEs confirmed by both proprietary classification algorithm and video verification)	164	0.59 (0.49-0.72)			

*Note: Impact count and impact rate per athlete exposure are presented for each data cleaning method. Impact rate ratios (Impact Rate for Method A divided by Impact Rate for Method B) and intraclass correlation coefficients (ICCs) with 95% confidence intervals (CI) were calculated to assess agreement between methods. ICCs were interpreted as follows: <0.5 = poor agreement, 0.5–0.75 = moderate agreement, 0.75–0.90 = good agreement, >0.90 = excellent agreement. * indicates a significant difference was observed. HAE = head acceleration event*

Table S2. Measures of validity across four data cleaning methods with video verification as the reference criteria.

Method Compared to Method 5 (Video-Verification)	Sensitivity	Specificity	Positive Predictive Value (PPV)
1 (Uncleaned)	1.00	0.00	0.03
2 (time-windowed HAEs only)	1.00	0.60	0.07
3 (algorithm-classified HAEs without time windowing)	0.33	0.97	0.26
4 (algorithm-classified HAEs with time windowing)	0.33	0.96	0.39
6 (HAEs confirmed by both proprietary classification algorithm and video verification)	1.00	N/A	1.00

Note: Sensitivity, specificity and positive predictive value (PPV) are presented for methods one through six. Method 5 (video-verification) is used as the reference criteria.

Table S3. Comparison of peak linear acceleration (PLA) across six data cleaning methods

Peak Linear Acceleration (PLA, g)					
**Method**	**Median**	**25th Percentile**	**75th Percentile**	**Compared Method**	**Corrected p-value**
1 (Uncleaned)	15.1	13.6	17.0	2	1.000
				3	1.000
				4	0.970
				5	1.000
				6	1.000
2 (time-windowed HAEs only)	15.0	13.5	16.7	3	1.000
				4	1.000
				5	1.000
				6	1.000
3 (algorithm-classified HAEs without time windowing)	13.9	12.6	16.7	4	1.000
				5	1.000
				6	1.000
4 (algorithm-classified HAEs with time windowing)	13.9	12.6	16.0	5	1.000
				6	1.000
5 (video verification)	14.4	13.0	17.3	6	1.000
6 (HAEs confirmed by both proprietary classification algorithm and video verification)	13.9	11.8	16.4		

Note: Median peak linear acceleration (PLA) values are presented for each data cleaning method. Differences in PLA across methods were assessed using a Related-Samples Friedman's Two-Way ANOVA with significance values adjusted by a Bonferroni correction.

Table S4. Comparison of peak rotational velocity (PRV) across six data cleaning methods

Peak Rotational Velocity (PRV, rad/s)					
**Method**	**Median**	**25th Percentile**	**25th Percentile**	**Compared Method**	**Corrected p-value**
1 (uncleaned)	8.5	6.5	10.5	2	1.000
				3	0.004 *
				4	0.294
				5	1.000
				6	1.000
2 (time-windowed HAEs only)	8.6	6.4	10.6	3	0.072
				4	1.000
				5	1.000
				6	1.000
3 (algorithm-classified HAEs without time windowing)	7.2	4.8	9.5	4	1.000
				5	0.777
				6	0.721
4 (algorithm-classified HAEs with time windowing)	7.3	5.3	10.1	5	1.000
				6	1.000
5 (video verification)	8.0	6.6	10.5	6	1.000
6 (HAEs confirmed by both proprietary classification algorithm and video verification)	7.5	5.6	9.1		

*Note: Median peak rotational velocity (PRV) values are presented for each data cleaning method. Differences in PRV across methods were assessed using a Related-Samples Friedman's Two-Way ANOVA with significance values adjusted by a Bonferroni correction. * indicates a significant difference was observed.*

The following tables (S5-S8) include data from <u>only the 9 boil-and-bite IMGs</u>.

Table S5. Comparison of head acceleration event (HAE) exposure across six data cleaning methods.

Method	Impact Number	Impact Rate (95% CI)	Compared Method	Impact Rate Ratio (95% CI)	ICC (95% CI)
1 (Uncleaned)	2398	582.19 (399.12-849.22)	2	2.81 (2.60-3.04) *	0.604 (0.022-0.891) *
			3	1199.00 (299.69-4797.02) *	-0.001 (-0.436-0.573) *
			4	1199.00 (299.69-4797.02) *	-0.001 (-0.436-0.573) *
			5	42.82 (32.85-55.81) *	-0.003 (-0.444-0.574)
			6	2398.00 (337.64-17031.14) *	0.000 (-0.435-0.573)
2 (time-windowed HAEs only)	854	246.89 (169.18-360.29)	3	427.00 (106.62-1710.15) *	-0.002 (-0.492-0.592)
			4	427.00 (106.62-1710.15) *	-0.002 (-0.492-0.592)
			5	15.25 (11.64-19.98) *	0.003 (-0.508-0.597)
			6	854.00 (120.16-6069.78) *	0.000 (-0.489-0.593)
3 (algorithm-classified HAEs without time windowing)	2	23.07 (15.70-33.92)	4	1.00 (0.14-7.10)	1.00
			5	0.04 (0.01-0.15) *	-0.009 (-0.524-0.569)
			6	2.00 (0.18-22.06)	0.014 (-0.622-0.640)
4 (algorithm-classified HAEs with time windowing)	2	15.56 (10.54-22.96)	5	7.50 (5.68-9.91) *	-0.009 (-0.524-0.596)
			6	420.00 (59.02-2988.68) *	0.014 (-0.622-0.640)
5 (video verification)	56	16.44 (11.15-24.25)	6	444.00 (62.40-3159.06) *	0.004 (-0.479-0.593)
6 (HAEs confirmed by both proprietary classification algorithm and video verification)	1	6.04 (4.02-9.07)			

*Note: Impact count and impact rate per athlete exposure are presented for each data cleaning method. Impact rate ratios (Impact Rate for Method A divided by Impact Rate for Method B) and intraclass correlation coefficients (ICCs) with 95% confidence intervals (CI) were calculated to assess agreement between methods. ICCs were interpreted as follows: <0.5 = poor agreement, 0.5–0.75 = moderate agreement, 0.75–0.90 = good agreement, >0.90 = excellent agreement. * indicates a significant difference was observed. HAE = head acceleration event*

Table S6. Measures of validity across four data cleaning methods with video verification as the reference criteria.

Method Compared to Method 5 (Video-Verification)	Sensitivity	Specificity	Positive Predictive Value (PPV)
1<br>(Uncleaned)	1.00	0.00	0.02
2<br>(time-windowed HAEs only)	1.00	0.66	0.07
3<br>(algorithm-classified HAEs without time windowing)	0.02	0.99	0.50
4<br>(algorithm-classified HAEs with time windowing)	0.37	0.96	0.39
6<br>(HAEs confirmed by both proprietary classification algorithm and video verification)	1	N/A	1

Note: Includes data from only the 9 boil-and-bite IMGs). Sensitivity, specificity and positive predictive value (PPV) are presented for methods one through six. Method 5 (video-verification) is used as the reference criteria.

Table S7. Comparison of peak linear acceleration (PLA) across six data cleaning methods

Peak Linear Acceleration (PLA, g)					
**Method**	**Median**	**25th Percentile**	**75th Percentile**	**Compared Method**	**Corrected p-value**
1 (Uncleaned)	15.8	15.1	22	2	1.000
				3	1.000
				4	1.000
				5	1.000
				6	1.000
2 (time-windowed HAEs only)	18.0	14.4	23.3	3	1.000
				4	1.000
				5	1.000
				6	1.000
3 (algorithm-classified HAEs without time windowing)	15.3	11.4	19.2	4	1.000
				5	1.000
				6	1.000
4 (algorithm-classified HAEs with time windowing)	15.3	11.4	19.2	5	1.000
				6	1.000
5 (video verification)	18.8	15.5	22.0	6	1.000
6 (HAEs confirmed by both proprietary classification algorithm and video verification)	11.4	11.4	11.4		

Note: Includes data from only the 9 boil-and-bite IMGs Median peak linear acceleration (PLA) values are presented for each data cleaning method. Differences in PLA across methods were assessed using a Related-Samples Friedman's Two-Way ANOVA with significance values adjusted by a Bonferroni correction.

Table S8. Comparison of peak rotational velocity (PRV) across six data cleaning methods

Peak Rotational Velocity (PRV, rad/s)					
**Method**	**Median**	**25th Percentile**	**25th Percentile**	**Compared Method**	**Corrected p-value**
1 (uncleaned)	9.2	8.0	13.5	2	1.000
				3	1.000
				4	1.000
				5	1.000
				6	1.000
2 (time-windowed HAEs only)	12.3	7.8	15.2	3	1.000
				4	1.000
				5	1.000
				6	1.000
3 (algorithm-classified HAEs without time windowing)	12.7	8.0	17.4	4	1.000
				5	1.000
				6	1.000
4 (algorithm-classified HAEs with time windowing)	12.7	8.0	17.4	5	1.000
				6	1.000
5 (video verification)	9.6	8.4	12.9	6	1.000
6 (HAEs confirmed by both proprietary classification algorithm and video verification)	8	8	8		

*Note: Includes data from only the 9 boil-and-bite IMGs Median peak rotational velocity (PRV) values are presented for each data cleaning method. Differences in PRV across methods were assessed using a Related-Samples Friedman's Two-Way ANOVA with significance values adjusted by a Bonferroni correction. * indicates a significant difference was observed.*
